# Gross Taper Failure and Fracture of the True Neck in Total Hip Arthroplasty: Retrieval Scanning Electron Microscope Analysis

**DOI:** 10.3390/medicina60030458

**Published:** 2024-03-09

**Authors:** Corrado Ciatti, Michelangelo Moschella, Edoardo Bori, Carlo Doria, Gianfilippo Caggiari, Bernardo Innocenti, Francesco Pisanu

**Affiliations:** 1 Department of Orthopaedics, Università degli Studi di Sassari, Viale San Pietro 24, 07100 Sassari, Italy; m.moschella@studenti.uniss.it (M.M.); cdoria@uniss.it (C.D.); gianfilippocaggiari@gmail.com (G.C.); pisanuf@gmail.com (F.P.); 2 BEAMS Department, Bio Electro and Mechanical Systems, École Polytechnique de Bruxelles, Université Libre de Bruxelles, Av. F. Roosevelt, 50 CP165/56, 1050 Bruxelles, Belgium; edoardo.bori@gmail.com (E.B.); bernardo.innocenti@ulb.be (B.I.)

**Keywords:** metallosis, gross trunnion fracture, Morse cone, SEM analysis, mechanically assisted crevice corrosion, revision surgery, retrieval SEM analysis, trunnionosis, fretting corrosion, implant fracture

## Abstract

*Background and objectives*: wear and corrosion can lead to the gross failure of the Morse taper junction with the consequent fracture of the true neck of the prosthetic stem in hip arthroplasty. *Materials and Methods*: 58-year-old male patient, with a BMI of 38 kg/m^2^. Because of avascular necrosis, in 2007, a metal-on-metal total hip arthroplasty was implanted in him, with a TMZF stem and a Co-Cr head. In December 2020, he complained of acute left hip pain associated with the deterioration of his left leg and total functional impairment, preceded by the crunching of the hip. X-rays and CT scan showed a fracture of the prosthetic neck that necessitated prosthetic revision surgery. A Scanning Electron Microscope (SEM) analysis of the retrieved prosthetic components was conducted. *Results*: Macroscopically, the trunnion showed a typical bird beak appearance, due to a massive material loss of about half of its volume. The gross material loss apparently due to abrasion extended beyond the trunnion to the point of failure on the true neck about half a centimeter distal from the taper. SEM analysis demonstrated fatigue rupture modes, and the crack began close to the neck’s surface. On the lateral surface, several scratches were found, suggesting an intense wear that could be due to abrasion. *Conclusions*: The analysis we conducted on the explanted THA showed a ductile rupture, began close to the upper surface of the prosthetic neck where the presence of many scratches had concentrated stresses and led to a fatigue fracture.

## 1. Introduction

Today, total hip arthroplasty (THA) is one of the most common joint replacement procedures worldwide [1,2]. Multiple factors are involved, including the progressive increase in people’s life expectancy and functional demands, along with a constant refinement of knowledge, both in surgical and biomedical engineering fields [3].

Nowadays, this surgical procedure guarantees excellent outcomes [4]. At the moment, THAs are designed with at least a femoral modularity, i.e., the head–neck system. This type of implant is based on the Morse-cone model, using the “cone on cone” concept [5]. It considers two conical elements pairing with intimate contact, guaranteeing a stable fixation through a circumferential compression. The two constituents are a male component defined as “trunnion” and a female one called “bore” [6].

The Morse-cone concept was ideated by Stephen A. Morse in 1864, and it was first applied in the mechanical field. Later, from the 1970s, it was also adopted for orthopedic surgery, significantly contributing to the evolution of total hip arthroplasty [5].

A further modularity, although optional, consists of the possibility to have a single stem design compatible with multiple necks to be assembled on the stem itself. The use of such modular femoral components shows multiple advantages: more precise restoration of patient’s anatomy, providing surgeons the chance to adapt component orientation during surgery; the possibility of correcting inferior limb heterometry, regulating offset and version and soft tissue tensioning; finally, making both the first surgical implant and the potential revision surgically easier [7]. On the other hand, as femoral necks and stems of these prosthetic models are separated elements, this leads to the presence of additional coupling interactions. This junction might be a spot with a greater risk of complications, even if more recent studies have shown that the responsibility for most of the failures related to these components is to be attributed to the materials (i.e., Co-Cr) rather than the prosthetic design [8,9,10].

In the literature, it has been demonstrated that a defective fitting is responsible for micromovements between surfaces, allowing the seepage of synovial fluid and the subsequent fretting corrosion [11,12]. This mechanism may lead to the increase in metal ion levels and debris inside the periprosthetic soft tissues, leading to both local and systemic complications [13]. At the joint level, the release of pro-inflammatory cytokines [14] and the correlated inflammatory reaction with lymphocytic aseptic infiltrate (ALVAL) [15], adverse reaction to metal debris (ARMD), and adverse local tissue reaction (ALTR) are frequently reported. In the worst cases, periprosthetic osteolysis is registered with the possible aseptic mobilization of the implant, the growth of pseudotumor masses, and even the complete fracture of the neck [16].

Numerous examples of metallosis related to metal-on-metal and metal-on-polyethylene implants have been documented in the literature. These cases are frequently linked to the development of pseudotumors and the emergence of adverse local tissue reactions; the prevalence of these issues in metal-on-metal hip arthroplasties was estimated to be between 36% and 61%. However, in the literature, a few cases of pseudotumors in patients with ceramic-on-ceramic couplings are also reported, although only case reports can be found [17].

In the case of prosthetic failure due to metallosis, the partial or total revision of the implant represents the main surgical solution [7,17,18]. In most cases, after the surgical revision of the prosthesis, patients report disappearance of pain, recovery of daily life activities, and improved range of motions [18].

The aim of this study is to analyze and report a failure model of the Morse taper junction with the consequent fracture of the true neck of the prosthetic stem.

## 2. Case Presentation

**Figure 1:**
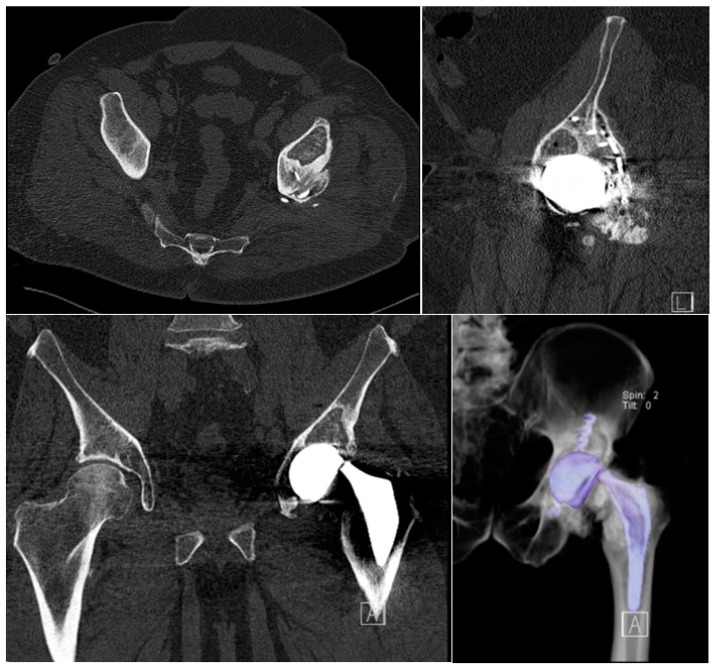
Preoperative CT scan.

We discuss here the case of a 58-year-old male patient, with a BMI of 38 kg/m^2^ and a weight of 110 kg, who was a former welder in the petrochemical industry (for 30 years) and then an agricultural entrepreneur. Silent anamnesis for significant issues was observed. In 2006, after a road accident, the patient underwent multiple surgeries including splenectomy, osteosynthesis for radius and ulna biosseous fracture, and open reduction and internal fixation (ORIF) of the posterior column of the left acetabulum, with plate and screws. In 2007, he underwent total left hip arthroplasty for avascular necrosis of the femoral head (metal-on-metal implant: ABG II monoblock stem in TMZF size 5, Stryker, Kalamazoo, MI, USA; MITCH TRH System acetabular component 54 mm and femoral head 48 mm + 0 in Co-Cr developed by Stryker Orthopaedics, in conjunction with Finsbury Orthopaedic Ltd., Leatherhead, UK). The postoperative radiographic evaluation showed the correct placement of the prosthetic components; the patient was discharged after 9 days of hospitalization without any complication and reported an excellent clinical outcome with complete return to regular activities of daily and working life. In December 2020, he began to feel the crunching of his left hip, which was not correlated to any functional impairment or pain; therefore, he underwent an outpatient orthopedic visit that suggested a radiographic evaluation. Unfortunately, because of the COVID-19 outbreak, this examination could not be booked in a short period and was booked 15 days after the consultation, and without any trauma explanation, he complained of acute left hip pain associated with the deterioration of his left leg and total functional impairment. He was admitted to the Emergency Room, and neurologic and vascular examinations were normal, but the patient was unable to walk and exhibited the shortening and external rotation of the left limb: the radiographic evaluation showed the implant components to be osseointegrated, but a fracture of the prosthetic neck was detected. The patient was admitted to the Orthopedic Department of the University Hospital of Sassari for a revision surgery. The CT scan performed prior to the operation revealed the appropriate osseointegration of stem and cup into the bone, and the prosthetic revision surgery was performed the following day (Figure 1). The posterolateral approach was followed, and an extended trochanteric osteotomy (ETO) was performed to remove the well-fixed femoral stem. The ETO was synthesized with two stainless steel cables (Accord 2.0 mm, Smith & Nephew, London, UK), and the femoral stem was replaced with a long monoblock cementless tapered fluted stem (Redapt stem size 16 × 300 mm standard, Smith & Nephew), bypassing the proximal osteotomy as described in previous articles [17]. The acetabular cup appeared macroscopically well fixed and showed a mirror-polished inner articular surface. In agreement with the literature [19], considering the safety of a partial revision, the monoblock acetabular cup that was already implanted was retained, and a dual-mobility head (Polarcup System, Smith & Nephew) with an XLPE bearing was implanted, appropriately matched to the acetabular shell. The patient began the rehabilitation protocol and resumed deambulation on the first postoperative day.

### Scanning Electron Microscope (SEM) Analysis

A microscopic study of the retrieved prosthetic components was conducted at the 4MAT Laboratory at Université Libre de Bruxelles. The SEM observations were performed with a FEI QUANTA 200 3D (Bridge Tronic Global, Fountain Valley, CA, USA).

**Figure 2:**
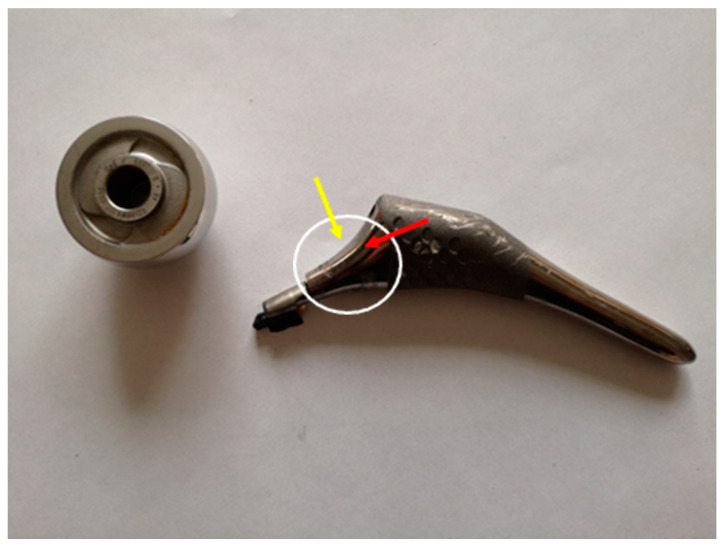
The broken femoral stem. The white circle highlights the fracture; the red arrow indicates the fracture surface; and the yellow arrow indicates the lateral surface.

Figure 2 shows that the rupture occurred in the neck in correspondence to the distal edge of the femoral head. This region (encircled in white in Figure 2 below) was analyzed using the SEM. In detail, both the fracture surface (red arrow) and the taper lateral surface (yellow arrow) of the neck were observed.

## 3. Results

### 3.1. Intraoperative Findings

During surgery, a massive fibro-cicatricial reaction with indistinct anatomical planes, small collections of dark greyish fluid and large areas of black granulation tissue, and massive periacetabular heterotopic calcifications were observed.

**Figure 3:**
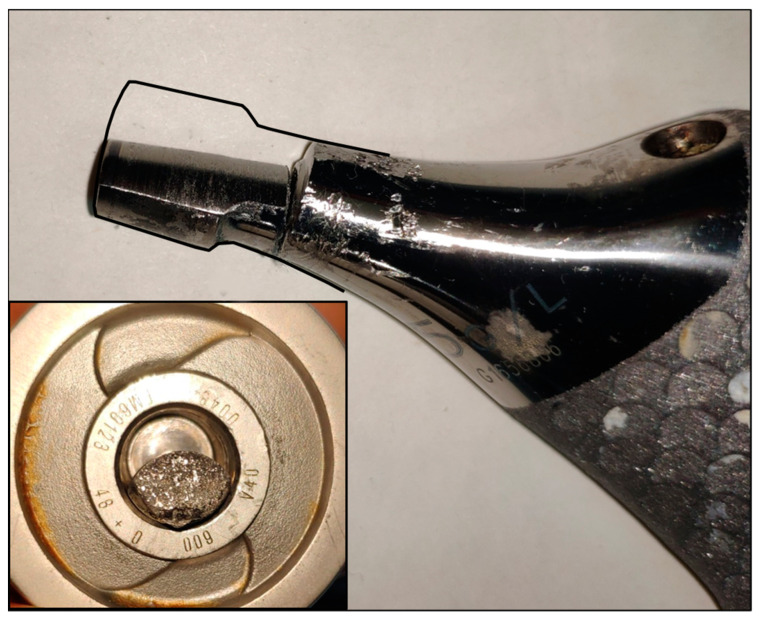
The profile of the intact trunnion is drawn with a black line, and the empty space is present between the trunnion and the bore in the small box, highlighting the gross material loss from the taper.

As indicated by the preoperative images, a well-fixed stem was found, and the fracture of the neck at the Morse taper junction was evident; the head was located inside the acetabular cup, and a metallic intra-articular loose body was then recognized to be the broken wear-distorted taper. On macroscopic observation, the trunnion showed a typical bird beak appearance, due to a massive material loss of about half of its volume. The gross material loss apparently due to abrasion extended beyond the trunnion to the point of failure on the true neck about half a centimeter distal from the taper (Figure 3).

### 3.2. Scanning Electron Microscope Results

#### 3.2.1. Fracture Surface

**Figure 4:**
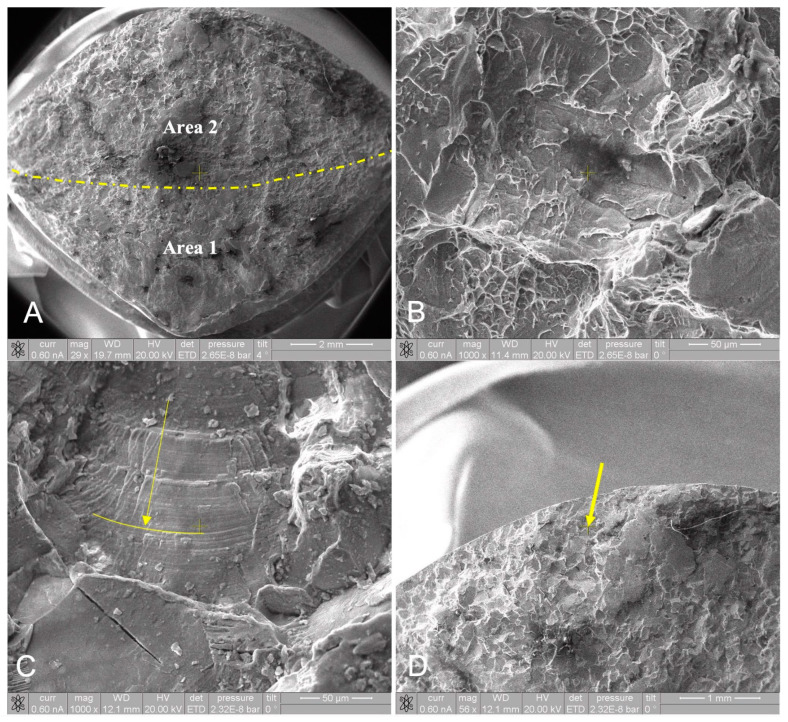
SEM of the fracture surface, and the scale is reported on the bottom right of each figure: (**A**) overview; the yellow dotted line divides Area 1 and Area 2; (**B**) SEM micrograph of Area 1; (**C**) SEM micrograph of Area 2, and the yellow line highlights the shape of the pattern of the fatigue fracture, and the yellow arrow indicates the fracture propagation direction; and (**D**) lower magnification of Area 2, already shown in figure (**C**) with a different scale.

Figure 4A depicts the low magnification of the fracture surface; the scale is also reported in the figure. Two areas (Area 1 and Area 2) can be distinguished based on their surface reliefs. The SEM micrographs of Area 1 and Area 2 are reported in Figure 4B,C. Area 1 clearly demonstrates the ductile fracture, deductible from the presence of dimples; by analyzing Area 2, the striations clearly indicate the behavior of fatigue rupture modes. The curvature of these striations display that the rupture has propagated in the direction of the yellow arrow (also shown on a low magnification micrograph of the same area in Figure 4D). This suggests that the crack began close to the neck’s surface. However, close inspection of this area revealed no particular defects.

Therefore, it was necessary to inspect the lateral face (yellow arrow on Figure 2) to check whether eventual defects could be found.

#### 3.2.2. Lateral Surface

**Figure 5:**
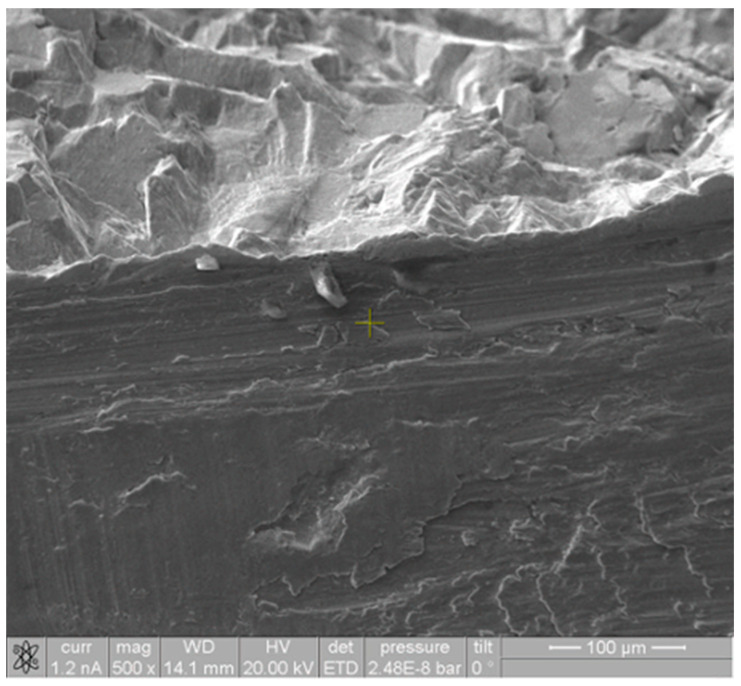
SEM of the lateral surface. The darker area in the bottom represents the actual fracture site, and the patterns corresponding to the scratches can be clearly identified in this region. However, the upper side of the image represents the trunnion lateral surface, on which no particular pattern was detected.

By inspecting the images of this area produced by the SEM, several scratches can be identified, suggesting intense wear that could be due to abrasion. Figure 5 illustrates the area around the edge between the lateral fracture surface and the trunnion surface.

## 4. Discussion

Although the Morse cone theoretically represents a high-locking mechanism, in the literature, micromovements are frequently reported, in particular in the case of not properly fitted components [7,16]. These micromovements allow the seepage of synovial fluid inside the junctional spaces, resulting in fretting corrosion and, subsequently, leading to local and general complications, even rarely including fatigue fracture of the prosthetic neck [12,16]. Considering that this phenomenon is often present in models with a single modularity, modular neck femoral stems seem to be even more likely to show this kind of issue as they present an additional component coupling (neck–head + stem–neck) [16]. Numerous studies documented cases of metallosis associated with modular neck implants, characterized by the elevated blood levels of chrome and cobalt in patients and the episodes of unfavorable local tissue reactions brought on by the release of metal particles. These events frequently resulted in the formation of a mass later, referred to as a pseudotumor. These issues have been linked to the use of a CoCrMo alloy in the production of modular necks. Actually, modularity is not a risk factor for metallosis, as shown by Maniscalco et al. in a significant number of patients with titanium modular neck hip arthroplasties [8]. In fact, they did not find any such complication in their cohort, nor any negative reaction in the surrounding tissue of the implants that would be associated with neck modularity. They came to the conclusion that the only thing that had prevented the occurrence of these events and the implant’s failure was the exclusive use of titanium necks rather than CoCrMo ones.

Neck fractures are complications documented in the clinical literature but not very common. In a recent systematic review, van Doesburg reported the results of 33 studies documenting a total of 80 prosthetic neck fractures of which 55 were in the head–neck region and 25 were more distal in the neck-shoulder region of the stem [12]. The fracture of the trunnion itself is an absolutely extraordinary event. In a case report, Botti et al. describe an atraumatic Co-Cr trunnion fracture within a long-skirted Co-Cr head [20].

In many cases, the main cause of implant failure is the so-called “trunnionosis” of the head–neck taper connection, defined as the contemporary presence of wear and corrosion [21,22]. This condition is due to a prolonged action of phenomena like wear and corrosion at the modular junction of the implant, particularly between head and neck, leading with time to a structural weakening of the prosthetic implant and also to a biological alteration of the neighboring soft tissue [22,23,24].

Lavernia et al., using a finite element model, found that the mechanical stresses on the trunnion and on the neck (in detail, in the area distal to the trunnion–head junction) were 8 times (for the trunnion) and 22 times (for the neck) greater than the stress exerted on a large-diameter femoral head, respectively [25]. The impairment of the trunnion, due to the high concentration of stress, might cause a fracture even without traumatic events [20,26]. The most striking cases of trunnionosis, often called gross trunnion failures (GTF), include fracture, the disassembly of the femoral head component, and a massive material loss from the trunnion [7,27]. Many situations can facilitate the occurrence of GTF, and the most important risk factors acknowledged in the literature are male sex, a body mass index (BMI) of >30, prosthetic implants with a high offset, long neck length or large CoCr femoral head (>36 mm), intraoperative damage of the components, and finally implant malpositioning [12,28].

Our case presents an atraumatic fracture of the stem at the neck–head junction, due to impaired mechanical properties following the intense abrasion of the β titanium alloy, known as the TMZF (Ti-12Mo-6Zr-2Fe) trunnion, by the large-diameter head made of Co-Cr. The SEM demonstrated a failure through a fatigue type of rupture. The final rupture was ductile (dimples in AREA 1). The initiation site was located close to the upper surface of the prosthetic neck. Intense abrasion on this surface produced deep scratches that may have led to stress concentrations sufficient to initiate the rupture. This intense abrasion in turn may have resulted from the contact between the neck and the femoral head. It is not possible a posteriori to say which mechanism triggered such an intense abrasion process. Wear at the in vivo trunnion interface is a complex process involving both mechanical and electrochemical processes.

The surface’s corrosion may play a fundamental role for the pathogenesis of this complication, and it can occur as a result of seepage of articular fluid among surfaces [11,12,16]. The cone turns into an anode and loses its oxide passivation layer once it touches the articular fluid, which has a low oxygen content [20,29]. This leads to a change in the physicochemical characteristics of the metal surface, with a consequent damage known as crevice corrosion [20,29,30]. Subsequently, there is dissolution of metals visible on its surface, and it is called galvanic corrosion [16,31]. In a retrospective study, Gilbert et al. analyzed 148 explanted THAs of both mixed (Ti-6Al-4V/Co-Cr) and similar (Co-Cr/Co-Cr) metal combinations, looking for signs of corrosion at the head–neck junction [30]. They reported higher percentages of corrosion in THAs with different metal coupling (35% of the heads and 16% of the necks) than those with the same metal coupling (23% of the heads and 14% of the necks), testifying how the prosthetic material plays a fundamental role in these phenomena [8]. Moreover, the TMZF was found to exhibit much more severe abrasive wear and tribo-chemical reactions in simulated body fluid than Ti-6Al-4V [32]. The accelerated wear of TMZF in simulated body fluid is attributed to its inability to resist the three-body abrasive wear due to its lack of strain-hardening capacity.

The correct coupling of the components is mandatory to minimize the risk of implant damage. For this reason, it is essential to avoid any possible defect created by surgical instruments on the component’s surface during the procedure and also avoid mixing head and stem components manufactured by different companies, because there is a major risk of procuring defective fitting, even if they appear perfectly assembled [5,7,12,20,29,33]. Furthermore, it is highly recommended to clean and dry the trunnion thoroughly before coupling it with the head [5,12,16,20,29]. As a matter of fact, in the case of the wet trunnion, it develops the anodic environment, which leads to crevice corrosion. On the other hand, damage on the surface creates iatrogenic scratches [12,20]. Finally, an effective pounding on the femoral head (a blow of 4 kN is generally recommended) ensures a more accurate fit [12,16].

### Limitations of the Study

Due to its status as a case report, the study’s primary weakness is its inability to produce data on rates, ratios, occurrences, or prevalences; future studies with larger cohort are required in order to precisely define this issue. Another significant constraint of the research is its retrospective design.

## 5. Conclusions

Gross trunnion fracture is a rare but possible event, usually caused by wear and corrosion at the head–neck taper junction. The analysis we conducted on the explanted THA showed a ductile rupture that began close to the upper surface of the prosthetic neck where the presence of many scratches concentrated stresses and led to a fatigue fracture.

Several forces act on the trunnion; thus, its impairment may lead to material loss, a non-traumatic fracture, and the disassembly of components. Micromovements are often the primum movens of fretting corrosion, as they allow the seepage of synovial fluids between prosthetic components. Subsequently, the taper loses its oxide passivation layer, and the so-called crevice corrosion starts, followed by galvanic corrosion.

To limit these phenomena, it is mandatory to avoid the damage of components during implantation, to avoid using components from different companies, to clean and dry the trunnion before coupling with the head, and to pound the head, creating a more accurate fit.

## Data Availability

The original contributions presented in the study are included in the article, and further inquiries can be directed to the corresponding author.

## References

[1] 2022 AOANJRR Annual Report and Supplementary Reports | New Zealand Orthopaedic Association. https://www.nzoa.org.nz/2022-aoanjrr-annual-report-and-supplementary-reports.

[2] Kremers H.M., Larson D.R., Crowson C.S., Kremers W.K., Washington R.E., Steiner C.A., Jiranek W.A., Berry D.J. (2015). Prevalence of Total Hip and Knee Replacement in the United States. J. Bone Jt. Surg. Am..

[3] Pisanu F., Andreozzi M., Costagli F., Caggiari G., Saderi L., Sotgiu G., Manunta A.F. (2020). Resumption of physical activity and sport after knee replacement. J. Orthop..

[4] Callaghan J.J., Albright J.C., Goetz D.D., Olejniczak J.P., Johnston R.C. (2000). Charnley total hip arthroplasty with cement. Minimum twenty-five-year follow-up. J. Bone Jt. Surg. Am..

[5] Hernigou P., Queinnec S., Flouzat Lachaniette C.H. (2013). One hundred and fifty years of history of the Morse taper: From Stephen A. Morse in 1864 to complications related to modularity in hip arthroplasty. Int. Orthop..

[6] Munir S., Walter W.L., Walsh W.R. (2015). Variations in the trunnion surface topography between different commercially available hip replacement stems. J. Orthop. Res..

[7] Banerjee S., Cherian J.J., Bono J.V., Kurtz S.M., Geesink R., Meneghini R.M., Delanois R.E., Mont M.A. (2015). Gross trunnion failure after primary total hip arthroplasty. J. Arthroplast..

[8] Maniscalco P., Quattrini F., Ciatti C., Ghidoni L., Ghidoni G., Burgio V., Pogliacomi F., Vaienti E., Ceccarelli F. (2020). Neck modularity in total hip arthroplasty: A retrospective study of nine hundred twenty-eight titanium neck implants with a maximum follow-up of eighteen years. Int. Orthop..

[9] Gofton W.T., Illical E.M., Feibel R.J., Kim P.R., Beaule P.E. (2017). A single-center experience with a titanium modular neck total hip arthroplasty. J. Arthroplast..

[10] Ollivier M., Parratte S., Galland A., Lunebourg A., Argenson J.N. (2015). Are titanium-on-titanium TiAl6V4 modular necks safe in total hip arthroplasty for non-overweight patients? Results of a prospective series at a minimum follow-up of 7 years. Eur. J. Orthop. Surg. Traumatol..

[11] Hallab N.J., Messina C., Skipor A., Jacobs J.J. (2004). Differences in the fretting corrosion of metal-metal and ceramic-metal modular junctions of total hip replacements. J. Orthop. Res..

[12] van Doesburg P.G., van Langelaan E.J., Apachitei I., Bénard M.R., Verdegaal S.H.M. (2020). Femoral prosthesis neck fracture following total hip arthroplasty—A systematic review. Arthroplasty.

[13] Kretzer J.P., Mueller U., Streit M.R., Kiefer H., Sonntag R., Streicher R.M., Reinders J. (2018). Ion release in ceramic bearings for total hip replacement: Results from an in vitro and an in vivo study. Int. Orthop..

[14] Oliveira C.A., Candelária I.S., Oliveira P.B., Figueiredo A., Caseiro-Alves F. (2014). Metallosis: A diagnosis not only in patients with metal-on-metal prostheses. Eur. J. Radiol. Open.

[15] Willert H.G., Buchhorn G.H., Fayyazi A., Flury R., Windler M., Köster G., Lohmann C.H. (2005). Metal-on-metal bearings and hypersensitivity in patients with artificial hip joints. A clinical and histomorphological study. J. Bone. Jt. Surg. Am..

[16] Pansard E., Fouilleron N., Dereudre G., Migaud H., Girard J. (2012). Severe corrosion after malpositioning of a metallic head over the Morse taper of a cementless hip arthroplasty. A case report. Orthop. Traumatol. Surg. Res..

[17] Pisanu F., Andreozzi M., Fiori E., Altamore F., Bartoli M., Caggiari G., Ortu S., Rios M., Manunta A.F., Doria C. (2021). Surgical management of hip prosthetic failure in metallosis: A case series and literature review. J Orthop..

[18] Ciatti C., Maniscalco P., Bosio S., Pagliarello C.P., Bianchi G., Quattrini F. (2023). Pseudotumor from ceramic-on-ceramic total hip arthroplasty. Int. J. Surg. Case Rep..

[19] Colacchio N.D., Wooten C.J., Martin J.R., Masonis J.L., Fehring T.K. (2020). Dual Mobility for Monoblock Metal-on-Metal Revision-Is It Safe?. J. Arthroplast..

[20] Botti T.P., Gent J., Martell J.M., Manning D.W. (2005). Trunnion fracture of a fully porous-coated femoral stem. Case report. J. Arthroplast..

[21] Rieker C.B., Wahl P. (2020). What the Surgeon Can Do to Reduce the Risk of Trunnionosis in Hip Arthroplasty: Recommendations from the Literature. Materials.

[22] Mistry J.B., Chughtai M., Elmallah R.K., Diedrich A., Le S., Thomas M., Mont M.A. (2016). Trunnionosis in total hip arthroplasty: A review. J. Orthop. Traumatol..

[23] Spiegelberg B.G., Lanting B.A., Howard J.L., Teeter M.G., Naudie D.D. (2018). Surface integrity of polyethylene liners following trunnionosis of a dual modular neck total hip implant. Hip Int..

[24] Pisanu F., Doria C., Andreozzi M., Bartoli M., Saderi L., Sotgiu G., Tranquilli Leali P. (2019). Pleomorphic clinical spectrum of metallosis in total hip arthroplasty. Int. Orthop..

[25] Lavernia C.J., Iacobelli D.A., Villa J.M., Jones K., Gonzalez J.L., Jones W.K. (2015). Trunnion-Head Stresses in THA: Are Big Heads Trouble?. J. Arthroplast..

[26] Wylde C.W., Jenkins E., Pabbruwe M., Bucher T. (2020). Catastrophic failure of the Accolade I hip arthroplasty stem: A retrieval analysis study. Hip Int..

[27] Eachempati K.K., Dannana C.S., Apsingi S., Ponnala V.K., Boyapati G., Parameswaran A. (2020). Trunnion fracture of femoral prosthesis following a large metal-on-metal uncemented total hip arthroplasty: A case report. Arthroplasty.

[28] Bolarinwa S.A., Martino J.M., Moskal J.T., Wolfe M.W., Shuler T.E. (2019). Gross trunnion failure after a metal-on-polyethylene total hip arthroplasty leading to dissociation at the femoral head-trunnion interface. Arthroplast. Today.

[29] Jacobs J.J., Cooper H.J., Urban R.M., Wixson R.L., Della Valle C.J. (2014). What do we know about taper corrosion in total hip arthroplasty?. J. Arthroplast..

[30] Gilbert J.L., Buckley C.A., Jacobs J.J. (1993). In vivo corrosion of modular hip prosthesis components in mixed and similar metal combinations. The effect of crevice, stress, motion, and alloy coupling. J. Biomed. Mater. Res..

[31] Osman K., Panagiotidou A.P., Khan M., Blunn G., Haddad F.S. (2016). Corrosion at the head-neck interface of current designs of modular femoral components: Essential questions and answers relating to corrosion in modular head-neck junctions. Bone Jt. J..

[32] Yang X., Hutchinson C.R. (2016). Corrosion-wear of β-Ti alloy TMZF (Ti-12Mo-6Zr-2Fe) in simulated body fluid. Acta Biomater..

[33] Hohman D.W., Affonso J., Anders M. (2011). Ceramic-on-ceramic failure secondary to head-neck taper mismatch. Am. J. Orthop..

